# Functional imaging with dynamic quantitative oblique back-illumination microscopy

**DOI:** 10.1117/1.JBO.27.6.066502

**Published:** 2022-06-30

**Authors:** Paloma Casteleiro Costa, Bryan Wang, Caroline Elizabeth Serafini, Annie Bowles-Welch, Carolyn Yeago, Krishnendu Roy, Francisco E. Robles

**Affiliations:** a Georgia Institute of Technology, School of Electrical and Computer Engineering, Atlanta, Georgia, United States; b Georgia Institute of Technology and Emory University, Wallace H. Coulter Department of Biomedical Engineering, Atlanta, Georgia, United States; c Georgia Institute of Technology, Marcus Center for Therapeutic Cell Characterization and Manufacturing, Atlanta, Georgia, United States; d Georgia Institute of Technology, Nuclear & Radiological Engineering and Medical Physics Program, Atlanta, Georgia, United States

**Keywords:** microscopy, quantitative phase imaging, stem cells, dynamic, functional imaging, label-free

## Abstract

**Significance:**

Quantitative oblique back-illumination microscopy (qOBM) is a recently developed label-free imaging technique that enables 3D quantitative phase imaging of thick scattering samples with epi-illumination. Here, we propose dynamic qOBM to achieve functional imaging based on subcellular dynamics, potentially indicative of metabolic activity. We show the potential utility of this novel technique by imaging adherent mesenchymal stromal cells (MSCs) grown in bioreactors, which can help address important unmet needs in cell manufacturing for therapeutics.

**Aim:**

We aim to develop dynamic qOBM and demonstrate its potential for functional imaging based on cellular and subcellular dynamics.

**Approach:**

To obtain functional images with dynamic qOBM, a sample is imaged over a period of time and its temporal signals are analyzed. The dynamic signals display an exponential frequency response that can be analyzed with phasor analysis. Functional images of the dynamic signatures are obtained by mapping the frequency dynamic response to phasor space and color-coding clustered signals.

**Results:**

Functional imaging with dynamic qOBM provides unique information related to subcellular activity. The functional qOBM images of MSCs not only improve conspicuity of cells in complex environments (e.g., porous micro-carriers) but also reveal two distinct cell populations with different dynamic behavior.

**Conclusions:**

In this work we present a label-free, fast, and scalable functional imaging approach to study and intuitively display cellular and subcellular dynamics. We further show the potential utility of this novel technique to help monitor adherent MSCs grown in bioreactors, which can help achieve quality-by-design of cell products, a significant unmet need in the field of cell therapeutics. This approach also has great potential for dynamic studies of other thick samples, such as organoids.

## 1 Introduction

Quantitative phase imaging (QPI) has become a mainstay label-free technology for monitoring live cells and their growth without the need for exogenous labels or stains, which can alter their behavior and function.^1^^–^^3^ This technique reveals information about a sample’s optical path length, yielding access to cellular structures below a nanometer and a sample’s refractive index (RI) distribution, which is linearly proportional to the cellular dry mass.^4^ Additionally, dynamic and longitudinal studies of cells (and other thin, live organisms) are possible with this method, which provides insight into cell migration, proliferation, mass transport, and other metabolic and functional processes.^5^^,^^6^ Unfortunately, phase imaging methods are largely limited to the analysis of thin, transparent samples, such as monolayer (2D) cell cultures, severely limiting their overall utility in biomedicine.

**Fig. 1:**
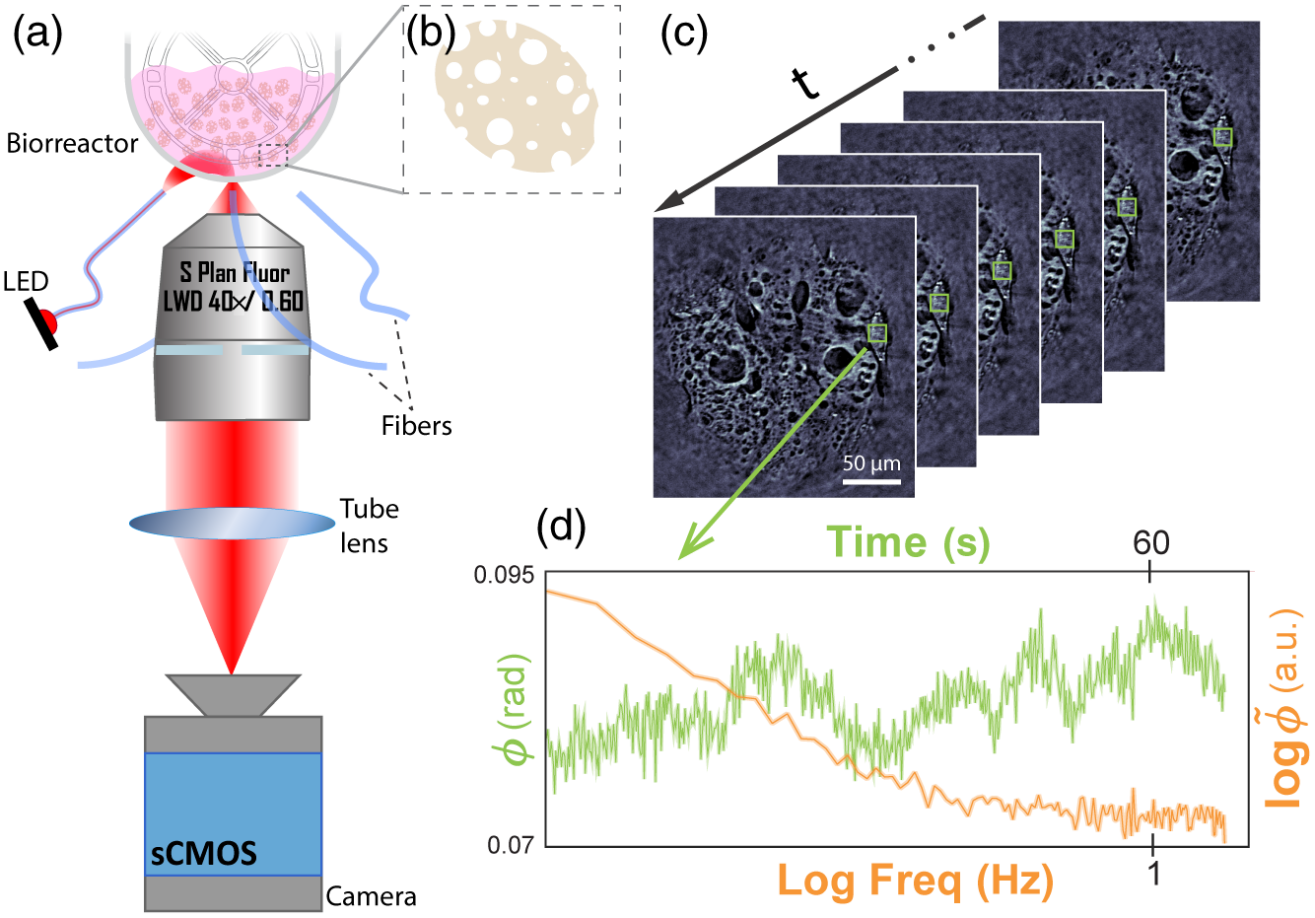
(a) qOBM system schematic. (b) Illustration of porous microcarrier (∼150 to 300  μm in diameter). (c) Timelapse qOBM stack of a microcarrier with adherent MSCs inside of a bioreactor. (d) Green: temporal phase value fluctuations from a single pixel corresponding to a cell region. Orange: log–log representation of the Fourier transform of the temporal phase value (green line).

To overcome this critical limitation to thin samples, we recently developed quantitative oblique back-illumination microscopy (qOBM), an epi-mode technique that provides 3D quantitative phase images of thick samples.^7^^,^^8^ This tomographic label-free, non-invasive, affordable, and real-time quantitative imaging technique has been applied to image the cellular and subcellular structures of samples such as tumors in fresh thick brain tissue, blood cells in collection bags, and thick organoids.^7^^–^^11^ Here, we propose dynamic-qOBM (DqOBM) to enable functional imaging based on the dynamics of the RI distribution of cellular and subcellular structures. In DqOBM, a sample is imaged over a period of time (e.g., fraction of a minute), and then each pixel is colorized based on the frequency response of its dynamic signal (see Fig. 1). The final DqOBM image is a functional map that represents cellular activity.

To show the utility of this method, we present an application of DqOBM to image 3D cell cultures on microcarriers used for cell expansion in bioreactors, which is critical for cell biomanufacturing of therapies and other biologics.^12^ Indeed, cell therapies have immense promise and transformative potential to treat a number of diseases,^13^ but our current inability to quantitatively monitor cell culture processes in-line, non-invasively, without labels, and using affordable and scalable methods has been a major limitation in biomanufactoring.^12^^,^^14^ Part of the challenge is that bioreactors are bulky, and microcarriers are highly scattering (i.e., optically opaque), effectively turning these systems into black boxes. Here, we show that DqOBM has the potential to monitor cells in these complex environments with a low-cost and highly accessible system, thus addressing a critical unmet need in cell and cell-derived biologics manufacturing.

## 2 Materials and Methods

As shown in Fig. 1(a), qOBM comprises a typical bright field microscope system, using sequential illumination from four light-emitting diodes (LEDs) (Luxeonstar, 720 nm) coupled into multimode fibers, positioned 90-deg from one another around the objective. When light from a single LED illumination enters the sample [as shown in Fig. 1(a)], photons undergo multiple scattering, causing some photons to change trajectory and effectively producing an oblique virtual light source within the sample. The photons that return within the angle of acceptance of the objective (Nikon S Plan Fluor LWD 40×, 0.6 NA) are then imaged onto an sCMOS camera (pco.edge 4.2 LT). The illumination wavelength (720 nm) was chosen due to the improved penetration depth (due to lower scattering), negligible-to-no phototoxicity, and high quantum efficiency of the camera for this spectral region. When two images from opposing illumination angles are subtracted, a differential phase contrast (DPC) image is obtained. DPC images provide qualitative details of phase differences along the direction of the sources, as previously introduced by Ref. 15. In qOBM, two orthogonal DPC images are acquired, and the data is quantified by deconvolving with the system’s optical transfer function via a Tikhonov regularized deconvolution following^7^^,^^8^^,^^16^

$$\phi =F^{-1}\{\frac{\underset{k}{\sum }{\overset{¯}{I}}_{\mathrm{DPC}}^{k}\cdot C_{\mathrm{DPC}}^{*}}{\underset{k}{\sum }{\left|C_{\mathrm{DPC}}\right|}^{2}+\alpha }\},$$ (1)

where $F^{-1}$ denotes the inverse Fourier transform, k=2 for the two orthogonal DPC images, ${\overset{¯}{I}}_{\mathrm{DPC}}^{*}$ is the Fourier transform of the k’th DPC image, α is the regularization parameter, and $C_{\mathrm{DPC}}^{*}$ is the complex conjugate DPC transfer function given as

$$C_{\mathrm{DPC}}=\frac{-i\cdot \int [S(u)-S(u^{\prime })]P(u+q)P^{*}(u)d^{2}u}{\int S(u)P(u)P^{*}(u)d^{2}u}.$$ (2)

Here, u is the 2D spatial frequency coordinates, and $u^{\prime }$ represents the same coordinates as u inverted along the shear direction. P represents the pupil function of the system, and S is the effective light source angular distribution at the focal plane, estimated through Monte Carlo photon transport simulation (performed with MCXLAB in MATLAB).^17^ It is important to note that this process in qOBM greatly improves image quality compared with differential phase contrast and provides quantitative phase information in thick samples. Further, like other non-interferometric phase imaging methods,^16^^,^^18^ qOBM^7^^,^^8^ does not suffer from π-wrapping artifacts. Information about absorption can also be obtained by summing the raw acquisitions (instead of subtracting as in DPC), but since the microcarriers and cells do not absorb the 720-nm illumination light, little to no information is conveyed in such absorption images.

In this work, we imaged live mesenchymal stromal cells (MSCs)^19^ adherent to porous microcarriers (∼300  μm in diameter)^20^ [Fig. 1(b)] using a 40× microscope objective with a 0.6 NA. The resolution of the system is ∼0.7  μm, limited by diffraction (this was confirmed experimentally using 300-nm polystyrene beads.). The MSCs were harvested from human umbilical cord tissue from a single, consent-signed, and de-identified donor. MSCs derived from human umbilical cord tissue provide important benefits for cell therapeutics due to their immunosuppressive and anti-inflammatory properties and their ability to differentiate into various types of cells.^10^^,^^21^ All isolation and early expansion procedures were performed by collaborators at the Marcus Center for Cellular Cures and approved by the Duke University Institutional Review Board.

To prepare MSCs on microcarriers, Cultispher G microcarriers were reconstituted in phosphate-buffered saline (PBS), sterilized, and stored in PBS at 4°C. Before seeding MSCs onto microcarriers, 70 mg of the microcarriers were extracted and re-suspended in 19 ml of MSC culture media (Prime XV XSFM). Next, 1.4 million passage-2 umbilical cord tissue MSCs (∼15  μm in diameter) were thawed and re-suspended in 1 ml of culture media, giving a $4000\text{ cells}/{\mathrm{cm}}^{2}$ seeding density with respect to the surface area of the microcarriers. The cell suspension was then combined with microcarriers and added to a PBS Mini 100-ml vessel (PBS Biotech). The vessel was placed on its base with a vertical-wheel impeller agitation rate of 20 revolutions per minute (RPM) in a humidified incubator set to 37°C and 5% ${\mathrm{CO}}_{2}$. The vessel was put on two 2-min agitation cycles with 30 min in between and then left static overnight to facilitate cell attachment. The following day, 50 ml of media was added to the vessel to reach a final volume of 70 ml. For the remainder of the cell expansion process (5 days), the agitation was continuous, starting at 30 RPM, increasing daily until 50 RPM was reached, and then maintaining at 50 RPM.

With the PBS Mini bioreactor, the vessel walls are thin (∼2  mm) and transparent, enabling monitoring of cells in-line, non-invasively, and continuously from outside the bioreactor, as shown in Fig. 1(a) (schematically) and 1C (experimentally). This same configuration can be applied to imaging other types of bioreactors from the outside (e.g., small to mid size vessels, clear bag bioreactors). However, for larger vessels (>1-l capacity), which are typically not made of transparent material, either the bioreactor or the microscope configuration would need to be adjusted. Here, for simplicity in this proof of concept study, the adherent MSCs on porous microcarriers were imaged at-line, that is, a small volume (∼1  ml) was pipetted from the bioreactor vessel atop a microscope slide and imaged directly without further processing. A single layer of microcarriers (∼300  μm in diameter) may not produce the necessary illumination through multiple scattering on its own to reconstruct a high SNR qOBM image. To overcome this challenge, we placed a polydimethylstyrene (PDMS) phantom with a high concentration of polystyrene beads with a well-characterized scattering profile to serve as an additional scattering layer over the sample.^22^^,^^23^ We simulated the illumination distribution of the system using the optical properties of the PDMS phantom. To capture the dynamic information, cells were imaged continuously at 4 or 32 frames per second (fps) over 1 or 8 min, respectively. Because four frames are required to reconstruct a qOBM image, capturing at 4 and 32 fps leads to a net qOBM imaging (i.e., phase imaging) rate of 1 and 8 Hz, respectively. The syncing was achieved through a Transistor-Transistor Logic (TTL) triggered by our custom imaging program for acquisition, coded in LabView.

**Fig. 2:**
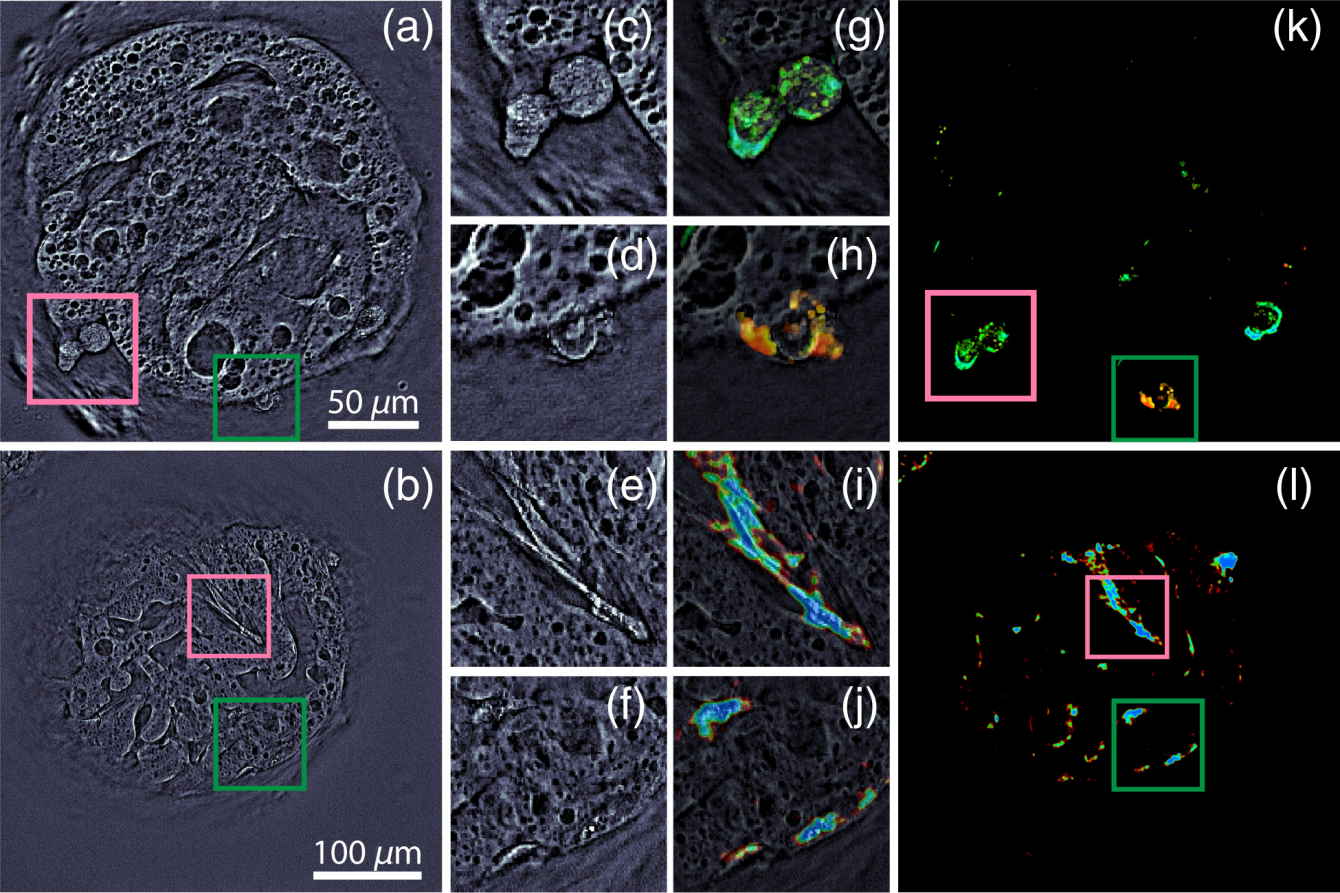
qOBM and DqOBM images of microcarriers surrounded by MSCs. (a) and (b) qOBM images of two microcarriers at 4 and 6 days of culturing, respectively. (c)–(f) Close-ups of pink and green regions in panels (a) and (b). (g)–(l) DqOBM functional images [(k) and (l)] and close-ups [(g)–(j)] of timelapses taken at [(g), (h), and (k)] 1 Hz over 8 min and [(i), (j), and (l)] 8 Hz over 1 min.

**Fig. 3:**
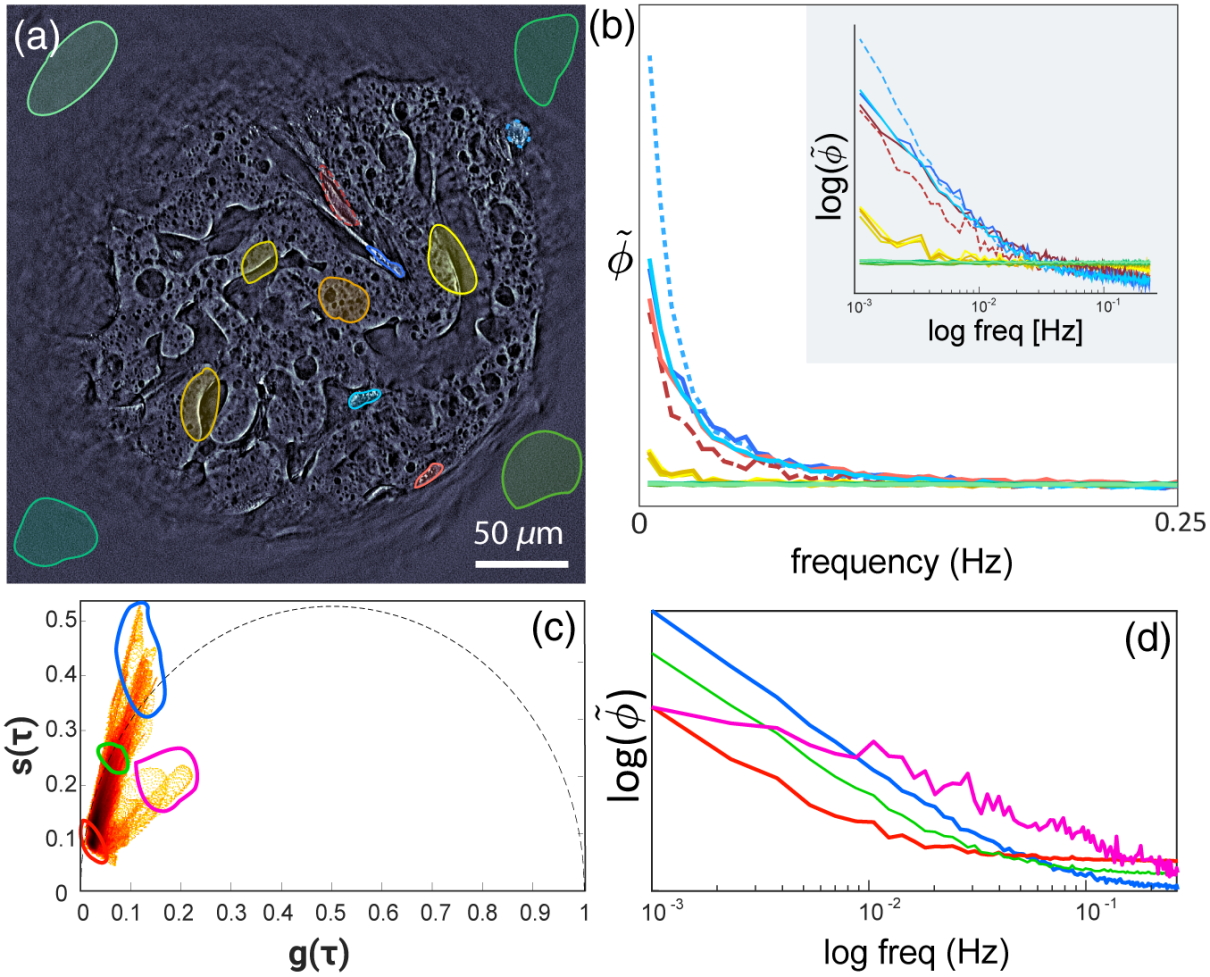
Phasor analysis. (a) qOBM image of microcarrier with adherent MSCs. ROIs in green correspond to background, yellow regions correspond to the static microcarrier, and red and blue regions correspond to live cells. (b) Average phase frequency response form selected ROIs in (a). Data acquired at 1 Hz over 8 min. (c) Cumulative phasor plot of ∼60 MSCs captured at 1 Hz over 8 min, mapped with τ=4  s (d) Log of the average signal responses of regions marked in (c).

Figures 1(c), 2(a), 2(b), and 3(a) show single qOBM images of microcarriers with adherent cells, which reveal clear structural details; however, the porous configuration of the microcarrier obscures the cellular structures. To increase the conspicuity of cells (for cell counting, for example) and enable phenotyping based on the functional behavior of cells, we leverage their dynamic behavior at the subcellular level. Although phase dynamics on the scale of many minutes to hours are linked to mass transport in the form of diffusive motion or advection (directed) motion in a region of interest (e.g., >10×10  μm), dynamics at the nano and micron scales (pixel-wise level) in time scales of several seconds have been linked to metabolic activity.^24^^,^^25^

Figure 3 illustrates the average dynamic frequency response from several representative regions inside and outside a microcarrier with adherent MSCs. It is worth noting that the dynamic frequency response is given by the absolute value of the Fourier transform of the temporal phase signal, $\tilde{\phi }(f)=|F\{\phi (t)\}|$, for each spatial pixel in the image, corresponding to $∼0.2\times 0.2\;\mu m^{2}$ with a cross-sectioning capability (z-resolution) of ∼2  μm [see Fig. 1(c)–1(d)]. Remarkably, the frequency response of MSCs appears mostly exponential [red and blue lines in Figs. 3(a) and 3(b) and orange line in Fig. 1(d)], indicative of subcellular mass movement that oscillates more strongly at low frequencies (i.e., longer-time scales) and dampens exponentially with increasing frequency. DqOBM reveals the subcellular dynamics of MSCs with an exponential slow component of >100  s ($<10^{-2}\;\mathrm{Hz}$) and a faster component of <100−1  s ($>10^{-2}-1\;\mathrm{Hz}$). Such functional behavior is expected for cell structures such as cell membranes^26^ and mitochondria,^27^ among other structures.^28^ As expected, the dynamic response in regions corresponding to the microcarrier without MSCs and background regions [green and yellow lines in Figs. 3(a) and 3(b)] show a mostly flat near-zero amplitude dynamic response, indicative of static behavior.

To visualize the cell dynamics, we apply phasor analysis,^29^^,^^30^ which is a natural choice given the exponential behavior of the dynamic phase frequency response $\tilde{\phi }(f)$. Phasor analysis is a common technique used to analyze signals based on their spectral/dynamic response (particularly exponential signals, such as in fluorescent lifetime and pump-probe microscopy).^29^^,^^30^ In phasor analysis, signals are decomposed into two variables, commonly named g and s, obtained by calculating the cosine and sine transforms (i.e. real and imaginary parts of the Fourier Transform) of the dynamic signals [here $\tilde{\phi }(f)$] for each spatial pixel in the image at a particular period, τ. Here, we choose τ=4  s or 0.5 s depending on the net acquisition rate (1 or 8 Hz, respectively) to decompose the signals into g and s following Eqs. (3) and (4), respectively:

$$g_{i}(\tau )=\frac{\int {\overset{˜}{\phi }}_{i}(f)\cos(2\pi f\tau )df}{\int {\overset{˜}{\phi }}_{i}(f)df},$$ (3)

$$s_{i}(\tau )=\frac{\int {\overset{˜}{\phi }}_{i}(f)\sin(2\pi f\tau )df}{\int {\overset{˜}{\phi }}_{i}(f)df}.$$ (4)

The two components, g and s, serve as coordinates in the phasor space and, i in Eqs. (1) and (2), represent the i’th pixel. Accordingly, each pixel in the image has a corresponding g and s value, and the phasor plot is a 2D histogram of these values. Signals with similar dynamics cluster together in phasor space, and mixtures between dynamic exponential signals map linearly from one region to another [Figs. 3(c) and 3(d)]. The endpoints of these distributions are referred to as endmembers.

A cumulative phasor plot of DqOBM signals from ∼60 MSCs is shown in Fig. 3(c). The net acquisition rate here was 1 Hz, and τ=4  s to provide the widest separation of signals from cells in phasor space. Dynamic signals with a low amplitude and slow response, corresponding to the background and microcarrier regions without cells, were omitted from the cumulative cell phasor plot. This was accomplished by segmentation using a prior mapping onto phasor space with τ=2.6  s or τ=0.33  s (for 1 and 8 Hz, respectively), which yields a clear separation between cellular structures and background.

## 3 Results and Discussion

The cumulative phasor plot [Fig. 3(c)] shows that signals mostly lie within the universal semicircle [black dotted line in Fig. 3(c)], which signifies that indeed the frequency response of the cellular dynamics follows a mostly exponential behavior,^29^^,^^30^ though phasors outside the universal semi-circle suggest that some dynamics may deviate slightly from a purely exponential frequency response. Figure 3(d) shows the average responses from four regions in phasor space [indicated by the blue, red, green, and magenta regions of interest (ROIs) in Fig. 3(c)], which again depict the signals’ mostly exponential or multiexponential behavior.

An important result in Fig. 3(c) is the presence of two continuous cluster distributions, branching off by the red ROI in the phasor space. This distribution is indicative of two distinct signal populations, each with varying dynamic responses. One population shows a dynamic distribution varying form the red ROI to the blue ROI, and the second spans a region in phasor space form the red ROI to the pink ROI. As Fig. 3(d) illustrates, the second population exhibits faster dynamics with a multiexponential behavior. There are no signals from the blue ROI to the magenta ROI, suggesting that the two populations are independent, that is, there is no mixture between these two end members, which indicates that these two signal populations correspond to either different cell phenotypes or unique subcellular components.

**Fig. 4:**
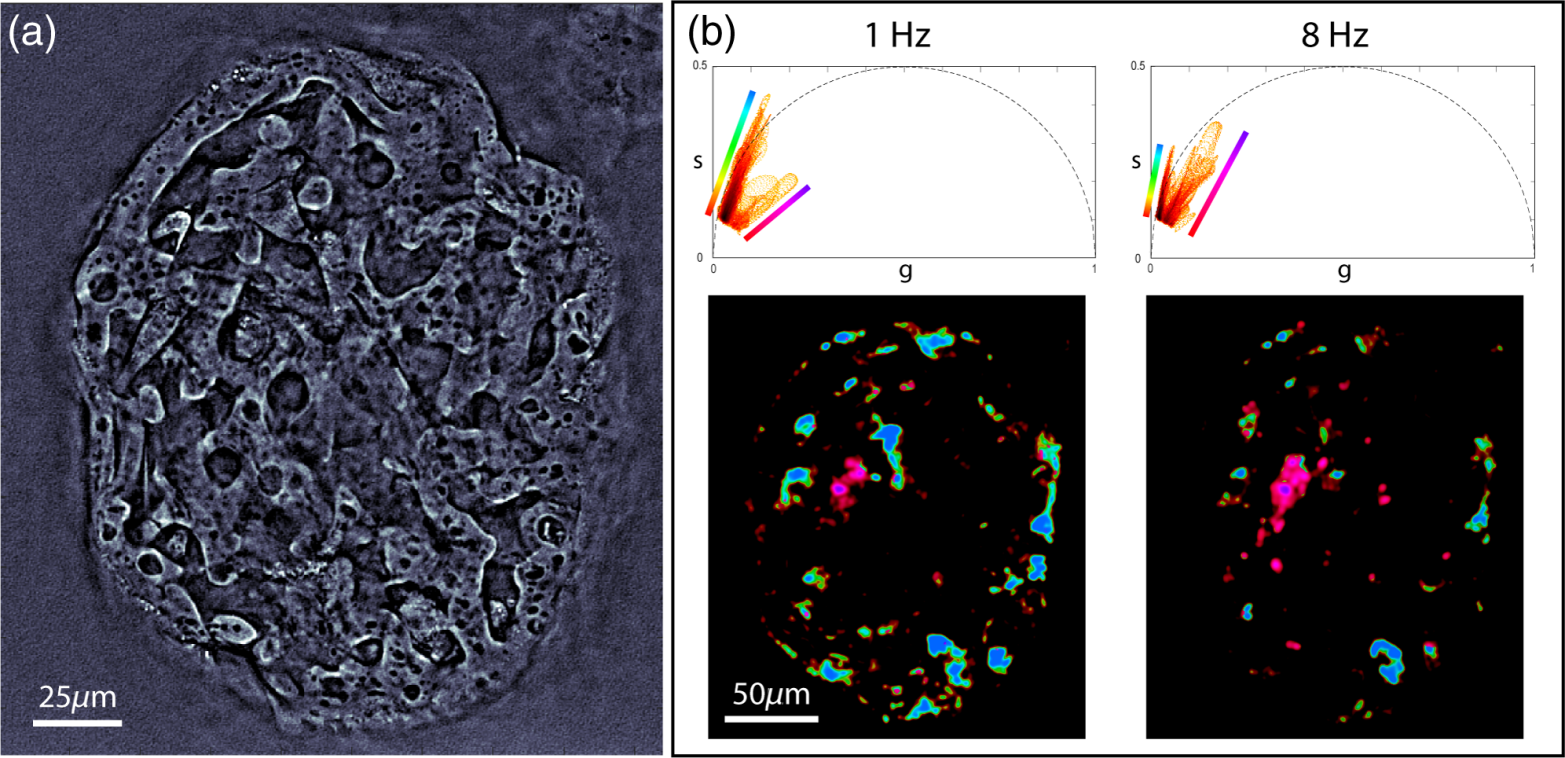
DqOBM captured at different imaging rates. (a) qOBM image of microcarrier with MSCs attached (b) Top: phasor plots of the same microcarrier imaged at 1 and 8 Hz, mapped with τ=4  s and τ=0.5  s, respectively. Bottom: corresponding DqOBM functional images. Videos 1 and 2 show each respective functional image overlayed over the qOBM timelapses. Color schemes for DqOBM are shown in the phasor plots (top) (Video 1, mov, 19.9 MB [URL: https://doi.org/10.1117/1.JBO.27.6.066502.1] and Video 2, mov, 25.9 MB [URL: https://doi.org/10.1117/1.JBO.27.6.066502.2]).

To further investigate the spatial distribution of these dynamic signals, we encode their location in phasor space with colors in the spatial images using an HSV color scheme. As illustrated in Figs. 4(a) and 4(b), slower signals are mapped to a red-to-yellow hue, whereas faster activity within the first population is given a green-to-blue hue. The second population follows a red-to-purple gradient to represent slower-to-faster dynamic signals. The value in the HSV color space is binary and set to 1 for cells and 0 otherwise, and the saturation is set to 1. The result of this mapping is a functional image where hue encodes dynamics.

Representative functional images are presented in Figs. 2 and 4. Figure 2 shows cells with dynamic signals that solely belong to the first population. Some cells show a fairly consistent slow dynamic response [Fig. 2(h)] or a moderate dynamic response [Fig. 2(g)], whereas others show a wider distribution [Figs. 2(i) and 2(j)]. The example provided in Fig. 4, however, shows the presence of signals from the second population, which clearly correspond to a unique cell type with higher dynamic activity, potentially indicating a more metabolically active cell. Although preliminary, these are important results showing the potential of DqOBM to identify distinct cell populations to help characterize cell expansion in these complex 3D structures.

Finally, we explore the dynamic behavior of the MSCs at different time scales. The imaging rate is currently limited by the camera (<40  Hz), making the net imaging rate limit 10 Hz. Figure 4 shows DqOBM functional maps of the same sample imaged at two different net qOBM frequencies of 1 and 8 Hz, over 8 and 1 min, respectively. As Fig. 4(b) shows, the DqOBM maps have strong similarities between the two acquisition rates. However, because of the extended imaging time of the first scan (8 min), small displacements (diffusive or advective) will also be detected, instead of just the fast fluctuations indicative of cellular activity. The slow displacement dynamics appear to improve the cell detection sensitivity, but the physical interpretation of the functional DqOBM image will differ. Also, we observe that regions belonging to the second (faster) signal population become more evident when imaging at higher rates, whereas the slower signals from the first population become harder to detect due to the shorter overall acquisition time. We note that long imaging times (>1  min) may not be practical in many settings and that shorter imaging times may provide dynamic maps that more faithfully represent metabolic activity.

Faster processes can be monitored using a faster camera. Although this does not appear to be necessary for MSCs, it may be desirable for other applications. It should be noted that we currently have ∼30  mW of power output from the multimode fibers, and the camera exposure time is ∼10  ms. Thus, simply switching to a faster camera would enable imaging up to 100 Hz without loss in the signal-to-noise ratio (SNR). Imaging rates can be increased further without loss in the SNR using higher power LEDs.

DqOBM shares some similarities with dynamic full-field optical coherence tomography (D-FFOCT), which was also recently developed for functional imaging.^25^ Both methods reveal subcellular RI dynamics indicative of cellular function. In fact, both show an exponential frequency response for temporal RI fluctuations of cells. However, DqOBM presents several advantages. First, any bright field microscope with a digital camera can be easily modified for qOBM and DqOBM at a low cost. D-FFOCT, on the other hand, requires a dedicated interferometer, typically in a Linnik configuration, which is difficult to align, susceptible to vibrations, and more complex and expensive. Furthermore, raw qOBM images have superior image quality (i.e., conspicuity to cellular and subcellular structures) compared with raw FF-OCT images, resulting from qOBM detecting the forward scatter field and OCT detecting the backscattered field. The former better retains an object’s low-frequency information and renders more natural images. However, D-FFOCT does show a deeper penetration depth (∼150  μm^25^ versus ∼100  μm^8^ with qOBM) resulting from the use of an external reference arm for coherence gating.

## 4 Conclusion

DqOBM enables functional imaging of optically thick samples based on subcellular dynamics using a simple and low-cost optical imaging system. The approach does not require complex equipment, including neither lasers nor delicate interferometers. The observed dynamics, which have previously been linked to metabolic activity, possess an exponential frequency response, which we analyze using phasor analysis. Phasor analysis not only allows us to colorize functional images (as with previously proposed methods^25^^,^^31^), but it also has the critical advantage of enabling a graphical representation of the dynamic signals, which can lead to a deeper understanding of different cell behaviors and function (e.g., via multiple end-member analysis, segmentation, etc.). Indeed, this graphical signal analysis was crucial in identifying the two signal populations that correspond to unique cell phenotypes of MSCs.

In this work, we have also applied DqOBM to monitor live adherent MSCs on porous microcarriers without labels. To the best of our knowledge, this is the first time such capabilities have been demonstrated. DqOBM can be integrated into bioreactors as a process analytical technology to enable real-time process monitoring, representing an important step towards generating highly effective cell products in a scalable, low-cost, and quality-by-design-driven manner. Thus, this method has the potential to address important challenges in cell manufacturing. Future work will focus on gaining a deeper understanding of how these dynamic signals correlate to cell metabolism, various cell phenotypes, and importantly, endpoints of interest in cell manufacturing processes. Nevertheless, the ability to clearly visualize cells in these complex structures is intrinsically significant. Furthermore, DqOBM can be broadly used for functional imaging in many other applications, including organoids, *in-vivo* tissue imaging, and more.^25^^,^^32^ Imaging of faster dynamics, which may be more critical in other applications, can be achieved using cameras with higher frame rates. Thus, we expect DqOBM to become an essential and accessible functional imaging tool.

## Supplementary Material





## Code, Data, and Materials Availability

Data and code underlying the results presented in this paper are not publicly available at this time but may be obtained from the authors upon reasonable request.
